# Risk of severe COVID-19 disease with ACE inhibitors and angiotensin receptor blockers: cohort study including 8.3 million people

**DOI:** 10.1136/heartjnl-2020-317393

**Published:** 2020-07-31

**Authors:** Julia Hippisley-Cox, Duncan Young, Carol Coupland, Keith M Channon, Pui San Tan, David A Harrison, Kathryn Rowan, Paul Aveyard, Ian D Pavord, Peter J Watkinson

**Affiliations:** 1 Primary Care Health Sciences, University of Oxford, Oxford, UK; 2 Adult Intensive Care Unit, John Radcliffe Hospital, Oxford, UK; 3 Kadoorie Centre, University of Oxford, Oxford, UK; 4 Division of Primary Care, School of Medicine, University of Nottingham, Nottingham, UK; 5 Division of Cardiovascular Medicine, British Heart Foundation Centre of Research Excellence, John Radcliffe Hospital, University of Oxford, Oxford, UK; 6 Nuffield Department of Primary Care Health Sciences, University of Oxford, Oxford, UK; 7 Clinical Trials Unit, ICNARC, London, UK; 8 Intensive Care National Audit and Research Centre ICNARC, London, UK; 9 Respiratory Medicine Unit and Oxford Respiratory NIHR BRC Nuffield Department of MedicineNDM Research BuildingOld Road CampusUniversity of Oxford, Oxford, UK; 10 Nuffield Department of Clinical Neurosciences, Oxford NIHR BRC, University of Oxford, Oxford, UK

**Keywords:** primary care, epidemiology, hypertension, diabetes, cardiac risk factors and prevention

## Abstract

**Background:**

There is uncertainty about the associations of angiotensive enzyme (ACE) inhibitor and angiotensin receptor blocker (ARB) drugs with COVID-19 disease. We studied whether patients prescribed these drugs had altered risks of contracting severe COVID-19 disease and receiving associated intensive care unit (ICU) admission.

**Methods:**

This was a prospective cohort study using routinely collected data from 1205 general practices in England with 8.28 million participants aged 20–99 years. We used Cox proportional hazards models to derive adjusted HRs for exposure to ACE inhibitor and ARB drugs adjusted for sociodemographic factors, concurrent medications and geographical region. The primary outcomes were: (a) COVID-19 RT-PCR diagnosed disease and (b) COVID-19 disease resulting in ICU care.

**Findings:**

Of 19 486 patients who had COVID-19 disease, 1286 received ICU care. ACE inhibitors were associated with a significantly reduced risk of COVID-19 disease (adjusted HR 0.71, 95% CI 0.67 to 0.74) but no increased risk of ICU care (adjusted HR 0.89, 95% CI 0.75 to 1.06) after adjusting for a wide range of confounders. Adjusted HRs for ARBs were 0.63 (95% CI 0.59 to 0.67) for COVID-19 disease and 1.02 (95% CI 0.83 to 1.25) for ICU care.

There were significant interactions between ethnicity and ACE inhibitors and ARBs for COVID-19 disease. The risk of COVID-19 disease associated with ACE inhibitors was higher in Caribbean (adjusted HR 1.05, 95% CI 0.87 to 1.28) and Black African (adjusted HR 1.31, 95% CI 1.08 to 1.59) groups than the white group (adjusted HR 0.66, 95% CI 0.63 to 0.70). A higher risk of COVID-19 with ARBs was seen for Black African (adjusted HR 1.24, 95% CI 0.99 to 1.58) than the white (adjusted HR 0.56, 95% CI 0.52 to 0.62) group.

**Interpretation:**

ACE inhibitors and ARBs are associated with reduced risks of COVID-19 disease after adjusting for a wide range of variables. Neither ACE inhibitors nor ARBs are associated with significantly increased risks of receiving ICU care. Variations between different ethnic groups raise the possibility of ethnic-specific effects of ACE inhibitors/ARBs on COVID-19 disease susceptibility and severity which deserves further study.

## Introduction

The first cases of infection caused by severe acute respiratory syndrome coronavirus 2 (SARS-CoV-2) (COVID-19) in the UK were confirmed on 24 January 2020. Since then the disease has spread rapidly through the population. There are no vaccines, preventative or curative treatments for COVID-19 disease and only one possible disease-modifying treatment^1^ so the government has used social distancing as a population-level intervention to limit the rate of increase in cases.

Case series of confirmed COVID-19 have identified age,^2^ sex,^3^ comorbidities^2 4^ and ethnicity^5^ as potentially important risk factors for susceptibility to infection, hospitalisation or death due to infection. In addition, chronic use of some medications at the time of exposure has been suggested as a potential risk factor for infection or severe adverse outcomes due to infection,^6^ although the evidence is currently too limited to confirm or refute these concerns.^7^ Understanding this chronic medication use is important because medications could be modified in individuals or at a population scale to alter the likelihood of infection or adverse outcomes. Furthermore, associations between medications and improved outcomes, if confirmed from large cohorts, could provide a basis for rapid prioritisation in prospective randomised clinical trials, and might provide important insights into disease mechanisms and pathogenesis.

SARS-CoV-1 and SARS-CoV-2, which have been responsible for the SARS epidemic and for the COVID-19 pandemic, respectively, interface with the renin-angiotensin-aldosterone system (RAAS) through ACE2, an enzyme that modulates the effects of the RAAS but is also the primary receptor for both SARS viruses. The interaction between the SARS viruses and ACE2 may be one determinant of their infectivity, and there are concerns that RAAS inhibitors may change ACE2 expression and hence COVID-19 virulence. This hypothesis has been extensively reviewed.^7^

ACE inhibitors and angiotensin receptor blocker (ARB) drugs are recommended by the National Institute for Health and Care Excellence as first-line treatment for patients under 55 years of age with hypertension and second-line treatment for those over 55 years of age and for those of African descent.^8^ ACE inhibitors are also widely used to treat congestive cardiac failure. Uncertainty around possible associations of these drugs with COVID-19 disease, and the subsequent risk that patients might stop taking these drugs of proven effectiveness, has led to regulatory and professional bodies issuing statements urging patients to keep taking their regular medications.^9^

Although several studies have considered the effect in hospitalised patients of drugs acting on the renin-angiotensin on disease course,^6 10 11^ none has looked at population use of these drugs to determine if they modulate susceptibility. We report a large, population-based study where we examined the drug histories of approximately 20% of all patients tested positive for coronavirus in England to determine if there was an independent association between ACE inhibitor and ARB drug prescription and severe COVID-19 disease susceptibility and progression.

## Methods

### Study design, sources of data and participants

We undertook a large, open cohort study of all patients aged 20–99 years registered with 1205 general practices in England contributing to the QResearch database (V.44, uploaded 24 March 2020) linked to COVID-19 RT-PCR test records (updated until 26 April 2020) and with intensive care records (updated until 27 April 2020). The protocol is published.^12^

### Primary care data and linked databases

We included general practices in England contributing to the QResearch database from which current data were available. QResearch is a high-quality research database established in 2002, which has been extensively used for pharmaco-epidemiological research.^13^ QResearch is the largest and most representative General Practitioner (GP) practice research database nationally.^14^

Two national databases were linked to QResearch. The first was the national registry of COVID-19 RT-PCR test positive results held by Public Health England (PHE). Since COVID-19 is a notifiable disease, laboratories in England are required to send results of all tests to PHE. At the time of analysis, 106 529 positive COVID-19 test results were available from 106 507 individuals in England, until 26 April 2020, of whom 104 665 were aged 20–99 years. Of these, 19 486 (18.6%) were linked to QResearch patients.

The second linked database was the Intensive Care National Audit and Research Centre (ICNARC) Case Mix Programme (CMP) database. This is a high-quality, clinical research database which includes contemporaneous data from 285 ICUs in England, Wales and Northern Ireland and is widely used for cohort studies, comparative audit and outcome data ascertainment for randomised clinical trials.^15 16^ As of 28 April 2020, there were 6968 patients admitted for ICU care with COVID-19 disease, of whom 6963 were aged 20–99 years. Of these, 1286 (18.5%) were linked to QResearch.

### Participants

We identified a cohort consisting of all patients aged 20–99 years who were fully registered with the GP practices on the start date (1 January 2020). Patients entered the cohort on this date and were censored at the earliest of the date of death, leaving the GP practice, the study end date (27 April 2020) or occurrence of the relevant outcomes of interest. We used all the relevant patients on the pooled database to maximise power and to enhance generalisability of the results.

### Outcomes

During our study period, over 98.6% of all COVID-19 RT-PCR tests in England were undertaken in a hospital setting for symptomatic patients sufficiently unwell to warrant hospital assessment and admission.

Our main outcomes for these analyses were:

1. COVID-19 RT-PCR test positive disease.
2. COVID-19-related admission for ICU care.

### Primary exposure variables

We had two main exposures of interest:

1. ACE inhibitors.
2. ARBs.

We classified a patient as having had exposure to either medication if they had three or more prescriptions, including a prescription issued in the 90 days preceding cohort entry.

### Explanatory variables

We extracted data from the GP record for explanatory and potential confounding variables including variables with some evidence of being risk factors for COVID-19 disease or severe disease as measured by ICU admission and variables likely to influence prescribing of ACE inhibitors and ARB medications. We used the latest information recorded in the GP record on or before study entry as follows:

1. Age (<40; 40–49; 50–59; 60–69; 70–79; 80+ years).
2. Ethnicity (nine categories—white and not recorded, Indian, Pakistani, Bangladeshi, other Asian, Black Caribbean, Black African, Chinese, other)
3. Deprivation quintiles (as measured by the Townsend score where quintile 1 is the most affluent and 5 is the most deprived).
4. Geographical region within England, categorised into 10 groups.
5. Body mass index (kg/m^2^), categorised into five categories—underweight (<20 kg/m^2^); normal weight (20–24.9 kg/m^2^); overweight (25–29.9 kg/m^2^); obese (30–34.9 kg/m^2^); severely obese (>35 kg/m^2^).
6. Smoking status in five categories—never-smoker; ex-smoker; light smoker (1–9 cigarettes/day); moderate smoker (10–19 cigarettes/day); heavy smoker (20+ cigarettes/ day).
7. GP recorded diagnosis of type 1 or type 2 diabetes.
8. GP recorded diagnosis of cardiovascular disease.
9. GP recorded diagnosis of congestive cardiac failure.
10. GP recorded diagnosis of hypertension.
11. GP recorded diagnosis of atrial fibrillation.
12. GP recorded diagnosis of asthma.
13. GP recorded diagnosis of chronic obstructive pulmonary disease.
14. GP recorded diagnosis of chronic kidney disease (CKD stage 3, 4 or 5).

We also extracted medication use for the following classes of drugs as potential confounding variables. We focused on classes of drugs rather than individual drugs to ensure adequate power. We classified patients as exposed using the same definitions as ACE inhibitors and ARBs.

1. Drugs to treat type 2 diabetes including sulfonylureas, biguanides and other drugs (thiazolidinediones, gliptins, sodium glucose co-transporter 2 inhibitors, glucagon-like peptide-1 receptor agonists, meglitinides).
2. Anticoagulant drugs (warfarin and direct oral anticoagulants).
3. Antiplatelet drugs.
4. Calcium channel blocking drugs.
5. Thiazides.
6. Potassium-sparing diuretics.
7. Statins.

### Statistical analyses

After conducting univariable analyses, we conducted a multivariable analysis based on patients with complete data. We then used multiple imputation with chained equations to replace missing values for ethnicity, body mass index and smoking status and used these values in our main analyses.^17^ We included all exposure and explanatory variables in the imputation model, along with the Nelson-Aalen estimator of the baseline cumulative hazard, and the outcome indicator. We carried out five imputations. We used Cox’s proportional hazards models to estimate adjusted HRs for ACE inhibitors and ARBs adjusting for the confounders. We tested for interactions between ACE inhibitors, ARBs and ethnicity.

We undertook several sensitivity analyses. To further reduce indication biases, additional analyses restricted to patients with hypertension or heart failure to directly compare risks for ACE inhibitors and ARBs with other antihypertensive drugs. We also undertook analyses adjusted for the number of antihypertensive drugs as a proxy for severity of hypertension (untreated hypertension; monotherapy; dual therapy; triple or more therapy). Lastly, we changed the definition of exposure to requiring a prescription within the last 30 days prior to cohort entry. We used p<0.01 (two-tailed) to determine statistical significance, to take account of multiple testing.

### Patient and public involvement

Patient representatives from the QResearch Advisory Board have advised the whether to undertake this research, on the data linkage, public interest and likely public benefit resulting from the study, dissemination of studies using QResearch data, including the use of lay summaries describing the research and its findings.

## Results

### Overall study population

One thousand two hundred five QResearch practices were included in our analysis. Of the 10 594 500 patients registered on 1 January 2020, 8 275 949 were aged between 20 and 99 years. Of these, 19 486 (0.24%) had a COVID-19 RT-PCR positive result and 1286 were admitted to an ICU.

### Baseline characteristics

Table 1 shows the baseline characteristics of the overall cohort consisting of 8 275 949 patients. The median age was 47 years (IQR 33–62); self-assigned ethnicity was recorded in 6 691 660 (80.9%). A total of 645 577 patients (7.8% of 8 275 949) were currently prescribed an ACE inhibitor and 308 881 (3.7%) were currently prescribed an ARB drug. Table 2 shows the proportions of patients prescribed ACE inhibitors and ARBs by ethnicity and other characteristics.

**Table 1:** Baseline characteristics of men and women aged 20–99 years registered with QResearch practices on 1 January 2020 and characteristics of patients with each of the two primary outcomes

Category	Total population (column %)	COVID-19 positive (column %)	COVID-19 ICU admission (column %)
Total population	8 275 949	19 486	1286
Male	4 115 973 (49.73)	9376 (48.12)	940 (73.09)
Age (years)			
Mean age (SD)	48.47 (18.41)	62.18 (20.84)	59.19 (12.52)
20–39	3 135 980 (37.89)	3487 (17.89)	95 (7.39)
40–49	1 399 562 (16.91)	2474 (12.70)	159 (12.36)
50–59	1 386 093 (16.75)	2927 (15.02)	366 (28.46)
60–69	1 037 077 (12.53)	2462 (12.63)	387 (30.09)
70–79	802 224 (9.69)	2734 (14.03)	242 (18.82)
80+ years	515 013 (6.22)	5402 (27.72)	37 (2.88)
Material deprivation			
Quintile 1 (most affluent)	1 877 761 (22.69)	3834 (19.68)	214 (16.64)
Quintile 2	1 819 942 (21.99)	3970 (20.37)	215 (16.72)
Quintile 3	1 671 924 (20.20)	4205 (21.58)	237 (18.43)
Quintile 4	1 490 725 (18.01)	3846 (19.74)	248 (19.28)
Quintile 5 (most deprived)	1 415 597 (17.10)	3631 (18.63)	372 (28.93)
Ethnicity recorded	6 691 660 (80.86)	16 379 (84.06)	1111 (86.39)
White/not recorded	6 960 062 (84.10)	14 976 (76.86)	788 (61.28)
Indian	228 467 (2.76)	847 (4.35)	66 (5.13)
Pakistani	147 397 (1.78)	399 (2.05)	48 (3.73)
Bangladeshi	110 368 (1.33)	256 (1.31)	44 (3.42)
Other Asian	146 174 (1.77)	661 (3.39)	70 (5.44)
Caribbean	93 949 (1.14)	557 (2.86)	59 (4.59)
Black African	199 200 (2.41)	812 (4.17)	106 (8.24)
Chinese	82 984 (1.00)	87 (0.45)	12 (0.93)
Other ethnic group	307 348 (3.71)	891 (4.57)	93 (7.23)
Geographical region			
East Midlands	216 535 (2.62)	238 (1.22)	13 (1.01)
East of England	296 236 (3.58)	562 (2.88)	19 (1.48)
London	2 080 923 (25.14)	6059 (31.09)	588 (45.72)
North East	194 027 (2.34)	600 (3.08)	26 (2.02)
North West	1 471 787 (17.78)	4042 (20.74)	220 (17.11)
South Central	1 104 114 (13.34)	2600 (13.34)	123 (9.56)
South East	927 208 (11.20)	1982 (10.17)	119 (9.25)
South West	899 722 (10.87)	1055 (5.41)	52 (4.04)
West Midlands	781 297 (9.44)	1759 (9.03)	95 (7.39)
Yorkshire and Humber	304 100 (3.67)	589 (3.02)	31 (2.41)
Smoking status			
Never smoker	4 745 455 (57.34)	12 036 (61.77)	791 (61.51)
Ex-smoker	1 774 275 (21.44)	5715 (29.33)	427 (33.20)
Light smoker	1 109 154 (13.40)	1102 (5.66)	47 (3.65)
Moderate smoker	213 629 (2.58)	155 (0.80)	7 (0.54)
Heavy smoker	98 748 (1.19)	97 (0.50)	2 (0.16)
Smoking not recorded	334 688 (4.04)	381 (1.96)	12 (0.93)
Body mass index (BMI)			
BMI <20 kg/m^2^	543 347 (6.57)	1076 (5.52)	13 (1.01)
BMI 20–24.99 kg/m^2^	2 438 268 (29.46)	4913 (25.21)	165 (12.83)
BMI 25–29.99 kg/m^2^	2 344 187 (28.33)	5925 (30.41)	410 (31.88)
BMI 30–34.99 kg/m^2^	1 090 042 (13.17)	3435 (17.63)	341 (26.52)
BMI 35+ kg/m^2^	619 487 (7.49)	2409 (12.36)	294 (22.86)
BMI not recorded	1 240 618 (14.99)	1728 (8.87)	63 (4.90)
Concurrent morbidity			
Chronic renal disease	338 693 (4.09)	3442 (17.66)	152 (11.82)
Asthma	1 089 645 (13.17)	2764 (14.18)	178 (13.84)
COPD	195 115 (2.36)	1421 (7.29)	46 (3.58)
Cardiovascular disease	433 631 (5.24)	3552 (18.23)	141 (10.96)
Atrial fibrillation	201 911 (2.44)	1870 (9.60)	41 (3.19)
Congestive cardiac failure	97 118 (1.17)	1211 (6.21)	25 (1.94)
Type 1 diabetes	39 094 (0.47)	208 (1.07)	23 (1.79)
Type 2 diabetes	536 516 (6.48)	4027 (20.67)	379 (29.47)
Hypertension diagnosis	1 414 021 (17.09)	7585 (38.93)	584 (45.41)
No medication	256 762 (3.10)	1804 (9.26)	93 (7.23)
Monotherapy	773 675 (9.35)	3754 (19.27)	249 (19.36)
Dual therapy	516 178 (6.24)	2540 (13.03)	215 (16.72)
Triple therapy	190 856 (2.31)	1005 (5.16)	119 (9.25)
Long-term medication			
ACE inhibitor	645 577 (7.80)	2864 (14.70)	266 (20.68)
ARB	308 881 (3.73)	1417 (7.27)	154 (11.98)
Beta-blockers	525 149 (6.35)	3185 (16.35)	170 (13.22)
Calcium channel blockers	654 171 (7.90)	3293 (16.90)	353 (27.45)
Other diabetes drugs	151 074 (1.83)	1183 (6.07)	148 (11.51)
Sulfonylureas	98 908 (1.20)	808 (4.15)	110 (8.55)
Biguanides	328 387 (3.97)	2135 (10.96)	262 (20.37)
Anticoagulants	207 061 (2.50)	1872 (9.61)	43 (3.34)
Antiplatelets	410 816 (4.96)	3049 (15.65)	172 (13.37)
Statins	1 073 039 (12.97)	5616 (28.82)	487 (37.87)
Thiazides	220 143 (2.66)	803 (4.12)	96 (7.47)
Potassium-sparing diuretics	46 825 (0.57)	417 (2.14)	11 (0.86)

Values are number (%) of patients unless indicated otherwise.

ARB, angiotensin receptor blocker; COPD, chronic obstructive pulmonary disease; ICU, intensive care unit.

**Table 2:** Numbers and proportions of patients taking ACE inhibitor or ARB medication according to patient characteristics

Category	Number in category	Prescribed ACE inhibitor (row %)	Prescribed ARB (row %)
Total population	8 275 949	645 577 (7.80)	308 881 (3.73)
Male	4 115 973	375 509 (9.12)	145 181 (3.53)
Female	4 159 976	270 068 (6.49)	163 700 (3.94)
Age (years)			
20–39	3 135 980	10 921 (0.35)	3635 (0.12)
40–49	1 399 562	44 117 (3.15)	14 746 (1.05)
50–59	1 386 093	125 971 (9.09)	46 885 (3.38)
60–69	1 037 077	163 430 (15.76)	74 343 (7.17)
70–79	802 224	176 435 (21.99)	95 393 (11.89)
80+	515 013	124 703 (24.21)	73 879 (14.35)
Material deprivation			
Quintile 1 (most affluent)	1 877 761	177 329 (9.44)	92 484 (4.93)
Quintile 2	1 819 942	161 223 (8.86)	80 220 (4.41)
Quintile 3	1 671 924	130 505 (7.81)	60 006 (3.59)
Quintile 4	1 490 725	101 993 (6.84)	44 660 (3.00)
Quintile 5 (most deprived)	1 415 597	74 527 (5.26)	31 511 (2.23)
Ethnicity			
White/not recorded	6 960 062	575 787 (8.27)	264 990 (3.81)
Indian	228 467	14 185 (6.21)	9936 (4.35)
Pakistani	147 397	10 198 (6.92)	5656 (3.84)
Bangladeshi	110 368	8189 (7.42)	4134 (3.75)
Other Asian	146 174	8000 (5.47)	5259 (3.60)
Caribbean	93 949	7478 (7.96)	5358 (5.70)
Black African	199 200	9379 (4.71)	6017 (3.02)
Chinese	82 984	1460 (1.76)	1112 (1.34)
Other ethnic group	307 348	10 901 (3.55)	6419 (2.09)
Geographical region			
East Midlands	216 535	14 004 (6.47)	6016 (2.78)
East of England	296 236	24 270 (8.19)	11 998 (4.05)
London	2 080 923	112 569 (5.41)	58 961 (2.83)
North East	194 027	17 597 (9.07)	6370 (3.28)
North West	1 471 787	137 043 (9.31)	61 705 (4.19)
South Central	1 104 114	89 462 (8.10)	42 668 (3.86)
South East	927 208	79 871 (8.61)	43 932 (4.74)
South West	899 722	75 767 (8.42)	32 850 (3.65)
West Midlands	781 297	70 613 (9.04)	33 605 (4.3)
Yorkshire and Humber	304 100	24 381 (8.02)	10 776 (3.54)
Smoking status			
Never smoker	4 745 455	335 769 (7.08)	181 411 (3.82)
Ex-smoker	1 774 275	227 398 (12.82)	103 363 (5.83)
Light smoker	1 109 154	62 039 (5.59)	18 364 (1.66)
Moderate smoker	213 629	11 542 (5.40)	3332 (1.56)
Heavy smoker	98 748	7929 (8.03)	2011 (2.04)
Smoking not recorded	334 688	900 (0.27)	400 (0.12)
Body mass index (BMI)			
BMI <20 kg/m^2^	543 347	13 050 (2.40)	5153 (0.95)
BMI 20–24.99 kg/m^2^	2 438 268	115 952 (4.76)	50 968 (2.09)
BMI 25–29.99 kg/m^2^	2 344 187	231 282 (9.87)	109 202 (4.66)
BMI 30–34.99 kg/m^2^	1 090 042	158 175 (14.51)	79 933 (7.33)
BMI 35+ kg/m^2^	619 487	108 568 (17.53)	55 489 (8.96)
BMI not recorded	1 240 618	18 550 (1.50)	8136 (0.66)
Concurrent morbidity			
Chronic renal disease	338 693	103 643 (30.60)	65 255 (19.27)
Asthma	1 089 645	83 948 (7.70)	4927 (4.52)
COPD	195 115	43 288 (22.19)	21 063 (10.80)
Cardiovascular disease	433 631	165 415 (38.15)	71 472 (16.48)
Atrial fibrillation	201 911	61 332 (30.38)	32 330 (16.01)
Congestive cardiac failure	97 118	43 746 (45.04)	21 637 (22.28)
Type 1 diabetes	39 094	8316 (21.27)	2989 (7.65)
Type 2 diabetes	536 516	193 155 (36.00)	88 308 (16.46)
Hypertension diagnosis	1 414 021	536 002 (37.91)	274 784 (19.43)
Monotherapy	773 675	230 565 (29.80)	105 921 (13.69)
Dual therapy	516 178	293 187 (56.80)	138 397 (26.81)
Triple therapy	190 856	121 825 (63.83)	64 563 (33.83)
Long-term medication			
ACE inhibitor	645 577		4119 (0.64)
ARB	308 881	4119 (1.33)	
Beta-blockers	525 149	189 691 (36.12)	86 126 (16.40)
Calcium channel blockers	654 171	241 203 (36.87)	119 143 (18.21)
Other diabetes drugs	151 074	65 933 (43.64)	29 245 (19.36)
Sulfonylureas	98 908	43 836 (44.32)	18 591 (18.80)
Biguanides	328 387	135 263 (41.19)	57 509 (17.51)
Anticoagulants	207 061	66 374 (32.06)	33 889 (16.37)
Antiplatelets	410 816	169 770 (41.33)	73 938 (18.00)
Statins	1 073 039	388 769 (36.23)	173 983 (16.21)
Thiazides	220 143	96 311 (43.75)	55 142 (25.05)
Potassium-sparing diuretics	46 825	20 660 (44.12)	11 807 (25.22_

ARB, angiotensin receptor blocker; COPD, chronic obstructive pulmonary disease.

**Figure 1:**
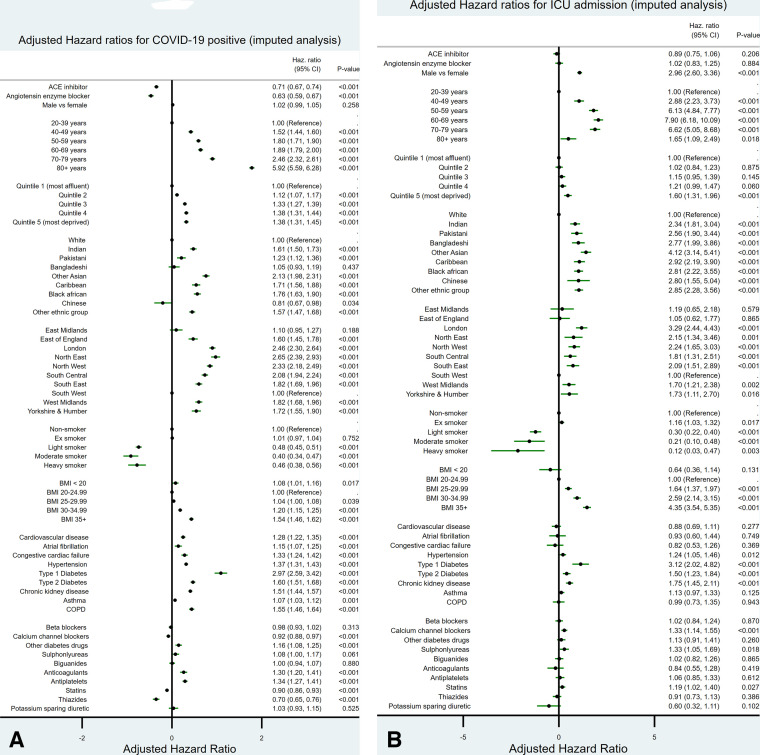
The adjusted HRs along with 95% CIs for (A) the outcome of a positive COVID-19 RT-PCR test and (B) the outcome of admission to an intensive care unit (ICU), for all the variables studied based on multiple imputed data. BMI, body mass index; COPD, chronic obstructive pulmonary disease.

**Figure 2:**
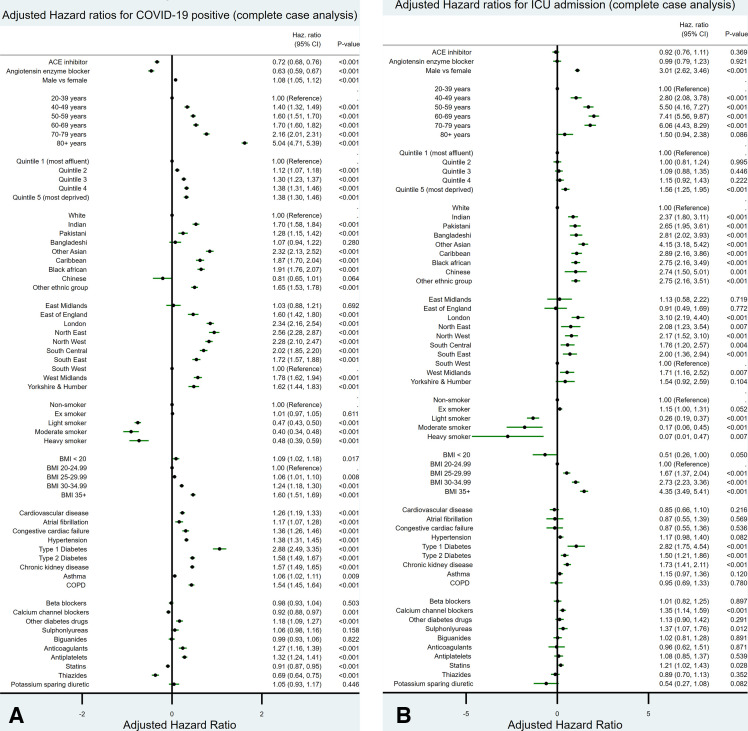
The adjusted HRs along with 95% CIs for (A) the outcome of a positive COVID-19 RT-PCR test and (B) the outcome of admission to an intensive care unit (ICU), for all the variables studied based on the completed case analysis. BMI, body mass index; COPD, chronic obstructive pulmonary disease.

Figure 1A and B show adjusted HRs for each outcome based on the multiply-imputed data. Figure 2A and B show the same for the complete case analysis.

### Associations of each outcome with the primary exposures of interest: ACE inhibitor and ARB medication

ACE inhibitors were associated with a significantly reduced risk of COVID-19 disease requiring hospital admission (adjusted HR 0.71, 95% CI 0.67 to 0.74) but were not significantly associated with risk of ICU care (adjusted HR 0.89, 95% CI 0.75 to 1.06) after adjusting for a wide range of confounders. Adjusted HRs for ARBs were 0.63 (95% CI 0.59 to 0.67) for COVID-19 disease and 1.02 (95% CI 0.83 to 1.25) for ICU care. The results were similar, when the proxy measure of hypertension severity was included with adjusted HRs of COVID-19 disease for ACE inhibitors of 0.87 (95% CI 0.72 to 1.05) and 0.82 (0.68 to 0.99) for ARB.

The results were similar when restricted to patients who had either hypertension or congestive cardiac failure. The adjusted HRs of COVID-19 disease requiring hospital admission associated with ACE inhibitors in this group was 0.69 (95% CI 0.65 to 0.73) and ICU admission was 0.96 (95% CI 0.78 to 1.16). The corresponding adjusted HRs for ARBs were 0.65 (95% CI 0.61 to 0.69) and 1.14 (95% CI 0.91 to 1.42).

There were significant interactions with ethnicity for ACE inhibitors and ARB (both p<0.001) for the COVID-19 RT-PCR diagnosed disease outcome. Table 3 shows the adjusted HRs for ACE inhibitor and ARB use for each of the ethnic groups. For ACE inhibitors the risks of COVID-19 disease were significantly higher in the Caribbean and Black African groups than the white group, with a significantly increased risk in the Black African group (adjusted HR 1.31, 95% CI 1.08 to 1.59), although the CIs were wide in the non-white ethnic groups. The risks associated with ARB use were significantly higher in the other Asian, Black African, Chinese and other ethnic group than the white group.

**Table 3:** Adjusted HRs (95% CI) for risk of COVID-19 positive test associated with ACE inhibitor and ARB exposure by ethnic group

	ACE inhibitor	P value	ARB	P value
	Adjusted HR (95% CI)		Adjusted HR (95% CI)	
White	0.66 (0.63 to 0.70)	<0.001	0.56 (0.52 to 0.62)	<0.001
Indian	0.74 (0.61 to 0.90)	0.003	0.66 (0.52 to 0.82)	<0.001
Pakistani	0.83 (0.64 to 1.09)	0.182	0.78 (0.57 to 1.06)	0.114
Bangladeshi	0.97 (0.72 to 1.31)	0.847	0.74 (0.49 to 1.13)	0.164
Other Asian	0.81 (0.64 to 1.03)	0.084	0.96 (0.73 to 1.23)	0.726
Caribbean	1.05 (0.87 to 1.28)	0.480	0.70 (0.53 to 0.92)	0.010
Black African	1.31 (1.08 to 1.59)	0.005	1.24 (0.99 to 1.58)	0.062
Chinese	0.73 (0.30 to 1.79)	0.575	1.53 (0.77 to 3.01)	0.223
Other ethnic group	0.82 (0.67 to 1.05)	0.122	1.09 (0.86 to 1.39)	0.475

HRs are comparing risks of COVID-19 in users versus non-users of ACE inhibitor and ARB, and are adjusted for age, sex, deprivation, geographical region, comorbidities (including hypertension included as a binary variable) and other medications listed in table 1.

ARB, angiotensin receptor blocker.

### Association of each outcome with age, sex, deprivation and ethnicity

While men were at no greater risk of having COVID-19 diagnosed disease requiring hospital admission than women (adjusted HR 1.02, 95% CI 0.99 to 1.05), they had a threefold increased risk of ICU admission despite adjustment for confounders (figure 1B). People from the most deprived areas had an increased risk of COVID-19 disease and ICU admission. There were regional variations in the risk of COVID-19 disease and ICU admission, the South West had the lowest risk of both outcomes, the North East had the highest risk of COVID-19 disease and London had the highest risk of ICU admission.

Overall, compared with the white ethnic group, all other ethnic groups except Chinese and Bangladeshi groups were associated with a significantly increased risk of COVID-19 disease. Highest risks were found for the other Asian group who had a 2.1-fold increased risk; Black African (1.8-fold increased risk); Black Caribbean (1.71-fold increased risk) and Indian (1.61-fold increased risk) compared with the white group. The comparative risk of ICU admission in these ethnic groups was even higher. Compared with the white group, all other ethnic groups had twofold to threefold higher risks of ICU admission, but smaller numbers of people in these groups led to some imprecise estimates.

### Association of each outcome with category of body mass index

The risks of COVID-19 disease and of ICU admission were higher in those with increasing BMI. The most pronounced gradient was for ICU admission, where being obese was associated with a 2.6-fold increased risk and severe obesity with a 4.4-fold increased risk compared with the normal weight group. This was after adjustment for all other variables shown in figure 1B.

### Association of each outcome with smoking status

There was a small increased risk of both adverse outcomes among ex-smokers compared with never-smokers. We observed a markedly decreased risk of both COVID-19 disease and ICU admission in smokers. The apparent protective association was greatest for heavy and moderate smokers and most markedly on the risk of ICU admission which was 88% lower in heavy smokers compared with non-smokers (figure 1B).

### Association of each outcome with comorbidity and concurrent medication

Each of the comorbidities included in the analysis was associated with an increased risk of COVID-19 disease. However, only CKD, hypertension, type 1 and type 2 diabetes were significantly associated with an increased risk of ICU admission.

Figure 1A shows significantly increased risks of COVID-19 disease associated with anticoagulants, antiplatelets, other diabetes drugs; significantly decreased risks of 10% for statins, 30% for thiazides and 8% for calcium channel blockers and no significant association for biguanides, beta-blockers or sulfonylureas. For ICU admission there was a significantly increased risk for calcium channel blockers, but no significant associations with the other drugs (at p<0.01).

## Discussion

### Summary of key results

In this very large population-based study, ACE inhibitor and ARB prescriptions were associated with a reduced risk of COVID-19 RT-PCR positive disease, having adjusted for a wide range of demographic factors, potential comorbidities and other medication. There was no evidence of an increased or reduced risk of ICU admission with either drug.

There were marked variations in risk of COVID-19 disease and of requiring ICU admission by ethnic group, with highest rates among Black, Asian, and minority ethnic (BAME) groups. This association is important and adds to existing knowledge^18^ since it is not explained by age, sex, deprivation, geographical region or several comorbidities and intercurrent medications included in our analysis.

### Comparisons with the literature

To date, published studies reporting associations between chronic medication with ACE inhibitor or ARB drugs and COVID-19 infections are limited to hospitalised patients^6 10 11 19^ or those attending a hospital clinic.^20^ This allows the study of drug treatment effects on the in-hospital disease course but not effects on disease susceptibility since there is no information on medication use in the uninfected or less severely infected population. Most in-hospital studies are relatively small containing low numbers of patients or ACE inhibitors of ARBs in comparison to our study. However, two^6 19^ were able to correct for the confounding effects of age, gender, comorbidities and in-hospital medications. In one study of 1128 patients with hypertension of whom 188 were taking ACE inhibitors/ARB,^6^ in-hospital use of ACE inhibitor or ARB medication was associated with a lower risk of all-cause in-hospital mortality (adjusted HR 0.42; 95% CI 0.19 to 0.92; p=0.03). In the larger 8910 patient study (770 taking ACE inhibitors and 556 ARBs), ACE inhibitors were associated with reduced in-hospital mortality (2.1% vs 6.1%; OR 0.33; 95% CI 0.20 to 0.54) but ARBs were not (6.8% vs 5.7%; OR 1.23; 95% CI 0.87 to 1.74).^19^ Conversely, there was no evidence of reduced risk in outcomes in patients receiving ACE inhibitor and ARB drugs in initial reports from New York.^11^

In our study, prior prescription of ACE inhibitor and ARB drugs did not have a significant effect on the risk of patients developing COVID-19 disease severe enough to require ICU care. In contrast, we found that previously prescribed ACE inhibitor and ARB drugs are associated with the likelihood of an individual testing positive for COVID-19 in a hospital setting. The effect was similar for both drug classes. This may indicate that drug treatment at the time of exposure altered susceptibility to COVID-19 infection and/or altered the likelihood of an infection progressing to the point where testing is sought. It is also possible that this reflects a ‘healthy user’ selection bias. There are no other population-based studies of ACE inhibitor/ARB use and COVID-19 infection. Losartan is already being tested in a clinical trial as a treatment of COVID-19 infection.^21^ Its efficacy may depend on the context in which it is tested. Since the recommendations for treatment of hypertension differ according to ethnic groups and age, we considered the possibility that these factors might contribute to the observed association between ACE inhibitor or ARB use and COVID-19 disease or severity. ACE inhibitors are recommended as first-line treatment for hypertension, whereas calcium channel blockers are recommended in patients of black ethnic origin.^8^ Indeed, there were significant interactions between ethnicity, ACE inhibitor and ARBs for COVID-19 disease. ARBs were significantly less protective in the other Asian, Black African, Chinese and other ethnic group than the white group. ACE inhibitors appeared less protective in the Caribbean than the white group and were associated with an increased risk of COVID-19 disease in the Black African group. This raises the possibility of ethnic-specific effects of ACE inhibitors/ARBs on COVID-19 disease susceptibility and severity or unmeasured confounding. However, as numbers were relatively small in the non-white ethnic groups so CIs were wide, caution is needed in interpreting these results.

Studies of patients hospitalised with COVID-19 have noted a greater than expected number of patients with hypertension,^2^ and hypertension appears to be a risk factor for more severe COVID-19 disease across many studies.^4^ In our study, hypertension was a risk factor for being tested positive for COVID-19 in a hospital setting independent of ACE inhibitor and ARB treatment, but was only modestly associated with likelihood of ICU admission. We found an expected association with obesity, with those who are obese or severely obese having higher risk of COVID-19 disease and ICU admission. However, we have reported a counterintuitive finding for smoking, with light, moderate and heavy smokers having a lower risk for both COVID-19 disease and ICU admission. One systematic review concluded on the basis of limited evidence either there is no difference in risk by smoking status or that there is an increased risk in smokers.^22^ However, our data are consistent with very low rates of smoking seen in patients presenting with COVID-19 in Wuhan^23^ and similar data from the USA^24^ and with the findings of a more limited analysis of patients with COVID-19 in France.^25^ This may reflect a general immunomodulatory effect, a mechanism that is thought to explain the lower incidence of sarcoidosis, extrinsic allergic alveolitis and ulcerative colitis in current smokers.^26 27^ Alternatively, smoking may cause increased ACE2 mRNA expression in human lung much as ACE inhibitors or ARBs are believed to, suggesting a possible common protective mechanism for severe COVID-19 disease.^28^ Additional possible mechanisms include a direct protective effect of nicotinic receptor stimulation^29^ or an association of smoking with another protective factor. This finding arose when including smoking status as a confounder and should be interpreted cautiously. Further studies are required to verify the apparent protective association, determine whether it is independent of other risk factors, and investigate potential mechanisms.

### Strengths

We have used two high-quality, established large validated research databases (QResearch and ICNARC CMP) and linked them to the national register of COVID-19 test results. Our study is observational with strengths and inherent limitations since the data were collected as part of routine NHS care. Key strengths include the use of high-quality, established validated databases, size, representativeness, lack of selection, recall and respondent bias. UK general practices have good levels of accuracy and completeness in recording clinical diagnoses and prescriptions and provide the ability to update analyses as data change over time.^30^ It is therefore likely to be representative of the population of England. It has good face validity since it has been conducted in the setting where most patients in the UK are assessed, treated and followed up. We have been able to adjust for a wide range of confounders based on detailed coded information recorded in the patients’ electronic medical record.

We restricted the sample for these analyses to only include patients with hypertension or heart failure so that all patients, whether treated with ACE inhibitors/ARBs or not, had the same indication for treatment. This is an important additional analysis as hypertension and heart failure themselves are associated with adverse COVID-19 outcomes, and this restricted analysis reduces their confounding effect and allows for a more direct comparison of the antihypertensive drugs in people with indications for their use. We also accounted for ethnicity and other confounding variables in this restricted analysis which could influence the selection of an antihypertensive treatment and also be associated with COVID-19 outcomes. Some systematic differences are still likely between patients who are treated and those who are not, such as severity of hypertension. We have carried out an additional analysis where we adjusted for a proxy measure of severity.

### Limitations

There may be some over-ascertainment of exposure to medication since our definition was based on issued prescriptions rather than dispensed medication. Our analyses focused on drug classes rather than individual drugs as there were insufficient cases to support an analysis at individual drug level. We have not investigated the relationship between the intensity and duration of exposure and the risk of disease in this early analysis. We investigated the more mechanistically likely and therefore immediately clinically important drug associations. Other drug classes can be investigated as numbers accrue. Data on community and care home deaths or deaths occurring within hospital but not in ICU are not yet available from Hospital Episode Statistics and Civil Registrations. Linkage of the GP data to national registries of outcome data, updated in near real time, will have minimised ascertainment bias relating to laboratory confirmed cases. However, there will be underascertainment of total COVID-19 cases due to the current absence of widespread systematic testing strategy in the UK, and due to false negative tests. As UK health policy during the study period confined testing for COVID-19 to hospitalised patients, our data focus on the incidence of more severe disease, rather than all cases, as most people with probable COVID-19 are not admitted to hospital. Some patients deemed to be at high risk of adverse outcomes of COVID-19 will have self-isolated during our study period to reduce their risk of contracting the virus and if effective, may result in a selection bias with such patients less likely to be become infected and subsequently admitted to hospital or ICU. Not all acutely unwell patients in hospital are admitted to ICU and this may result in a selection bias. Admission to ICU is limited to those who might benefit from this treatment and so varies on the basis of patient demographic and medical characteristics. Data on deaths in ICU were available to us but a significant proportion of patients admitted to an ICU were still being treated in an ICU and this varied by region as the pandemic spread. For this reason, ICU deaths were not included in the analysis. Further analyses of mortality will be undertaken once the relevant data (including out-of-hospital deaths) become available.

We have undertaken two new novel data linkages by linking QResearch to both COVID-19 test results and outcomes recorded on the ICNARC CMP data. This new linked data asset is a valuable resource for future research projects.

## Conclusion

In this very large population-based study, ACE inhibitor and ARB prescriptions were associated with a reduced risk of COVID-19 RT-PCR positive disease in a hospital setting adjusting for a wide range of demographic factors, potential comorbidities and other medication. There was no evidence of an increased or decreased risk associated with either drug for ICU admission. There are marked variations in risk of COVID-19 disease and ICU admission by ethnic group, with highest rates among BAME groups. The strength of this association is greater with the more severe outcome and is not explained by age, sex, deprivation, geographical region or several comorbidities and intercurrent medications included in the analysis. The counterintuitive finding of smokers having a lower risk of COVID-19 disease requiring hospital admission and ICU admission deserves further study.

**Key messages**

**What is already known on this subject?**

- There is uncertainty about the interaction of ACE inhibitor and angiotensin receptor blocker (ARB) drugs with COVID-19 disease susceptibility and disease severity.

**What might this study add?**

- In this very large population-based study, treatment with ACE inhibitor and ARB prescriptions is associated with a reduced risk of COVID-19 RT-PCR positive disease after adjusting for a wide range of variables.
- Neither ACE inhibitors nor ARBs are associated with increased risks of receiving ICU care for COVID-19 disease.
- There are significant interactions with ethnicity for ACE inhibitors and ARBs for COVID-19 disease with higher risks among the non-white ethnic groups particularly Black African patients compared with the white group, although the confidence intervals for some analyses are wide; this finding is important and adds to existing knowledge.

**How might this impact on clinical practice?**

- Neither ACE inhibitors nor ARBs are associated with increased risks of COVID-19 RT-PCR positive disease or of receiving ICU care for COVID-19 disease.
- Variations between different ethnic groups raise the possibility of ethnic-specific effects of ACE inhibitors/ARBs on COVID-19 disease susceptibility and severity which deserves further study.
